# TM4SF5 promotes metastatic behavior of cells in 3D extracellular matrix gels by reducing dependency on environmental cues

**DOI:** 10.18632/oncotarget.17644

**Published:** 2017-05-07

**Authors:** Dae-Geun Song, Gyu-Ho Lee, Seo Hee Nam, Jin-Gyu Cheong, Doyoung Jeong, Seo-Jin Lee, Cheol-Ho Pan, Jae Woo Jung, Hye-Jin Kim, Jihye Ryu, Ji Eon Kim, Somi Kim, Chang Yun Cho, Min-Kyung Kang, Kyung-Min Lee, Jung Weon Lee

**Affiliations:** ^1^ Department of Pharmacy, Research Institute of Pharmaceutical Sciences, College of Pharmacy, Seoul National University, 08826 Seoul, Korea; ^2^ Systems Biotechnology Research Center, Korea Institute of Science and Technology (KIST), Gangneung-si, 25451 Gangwon-do, Korea; ^3^ Interdisciplinary Program in Genetic Engineering, Seoul National University, 08826 Seoul, Korea; ^4^ Department of Life Science and Biotechnology, Shingyeong University, Gyeonggi-do, 18274, Korea

**Keywords:** 3D cell culture, TM4SF5, invasive foci, microenvironment, vasculogenic mimicry

## Abstract

Transmembrane 4 L six family member 5 (TM4SF5) is highly expressed in hepatocellular carcinoma tissues and enhances migration in two-dimensional environments. Here, we investigated how TM4SF5 is involved in diverse pro-metastatic phenotypes in *in vivo*-like three-dimensional (3D) extracellular matrix gels. TM4SF5-positive cells aggressively formed invasive foci in 3D Matrigel, depending on TM4SF5-mediated signaling activity, cytoskeletal organization, and matrix metallopeptidase (MMP) 2-mediated extracellular remodeling, whereas TM4SF5-null cells did not. The TM4SF5-null cells did, however, form invasive foci in 3D Matrigel following inhibition of Rho-associated protein kinase or addition of collagen I, suggesting that collagen I compensated for TM4SF5 expression. Similarly, TM4SF5-positive cells expressing vascular endothelial-cadherin formed network-like vasculogenic mimicry in 3D Matrigel and collagen I mixture gels, whereas TM4SF5-negative cells in the mixture gels displayed the network structures only upon further treatment with epidermal growth factor. The foci formation also required MMP2-mediated remodeling of the extracellular matrix. Co-cultures exhibited TM4SF5-positive or cancer-associated fibroblasts at the outward edges of TM4SF5-null cell clusters. Compared with TM4SF5-null cells, TM4SF5-positive cells in 3D collagen gels showed a more invasive outgrowth with dramatic invadopodia. These observations suggest that TM4SF5 plays roles in the promotion of diverse metastatic properties with fewer environmental requirements than TM4SF5-negative cells.

## INTRODUCTION

Metastatic cancer cells must communicate in a bidirectional manner with the extracellular environment, including the extracellular matrix (ECM), cytokines, growth factors, and neighboring cells [1]. Cellular behaviors can be influenced by the extracellular environment, and intracellular signaling activities can lead to remodeling of the extracellular microenvironment [2]. Depending on the extracellular cues, cancer cells may adopt different migratory or invasive morphologies to navigate flexible or rigid extracellular contexts [3]. Migration and invasion depend on the dynamic regulation of cell adhesion properties via efficient formation of invadopodia, which are cellular structures where cell-ECM adhesions occur and ECM proteases localize to resolve ECMs [4, 5].

Cells cultured *in vitro* on flat, two-dimensional (2D) substrates can differ considerably in their morphologies and cell adhesion properties from those grown in three-dimensional (3D) *in vivo*-like environments surrounded with ECMs [6, 7]. Embedding tumor cells in 3D Matrigel or collagen type I gels can thus allow dynamic behaviors that replicate the behavior of metastatic tumor cells traveling through the ECM-enriched stromal area [8]. Cell cultures in 3D ECM gel may thus allow exploration of the mechanisms of cancer metastasis and screening for anti-metastatic reagents [9].

Transmembrane 4 L six family member 5 (TM4SF5), a transmembrane glycoprotein belonging to the transmembrane 4 L six family, is highly expressed in diverse cancers including hepatic cancer [10]. TM4SF5 has four transmembrane domains, similar to the tetraspanins, and forms TM4SF5-enriched microdomains (T_5_ERMs). Within these microdomains, TM4SF5 forms protein-protein complexes with other membrane receptors, including integrins and growth factor receptors, leading to unique intracellular signal transduction pathways [11]. The expression of TM4SF5 can cause cytosolic p27^kip1^-mediated RhoA inactivation, leading to morphological changes that promote multilayer-growth and migration [12]. TM4SF5 expression in hepatocytes can activate focal adhesion kinase (FAK) for enhanced migration [13], proto-oncogene tyrosine protein c-Src for enhanced invasion [14], and signal transducer and activator of transcription 3 (STAT3) to promote immune escape [15]. These findings come primarily from 2D cell culture experiments. We were interested in exploring the metastatic roles of TM4SF5 in *in vivo*-like 3D gel systems surrounded with ECM. Therefore, in this study, we explored the specific involvement of TM4SF5 in complex metastatic behaviors using 3D gel systems with diverse extracellular components.

We found that TM4SF5 expression in 3D gel-embedded cells promoted the formation of invasive foci, invadopodia, and an endothelial-like network via the intracellular signaling activity of TM4SF5 and extracellular environmental cues.

## RESULTS

### TM4SF5-positive cells form invasive foci in 3D Matrigel

**Figure 1:**
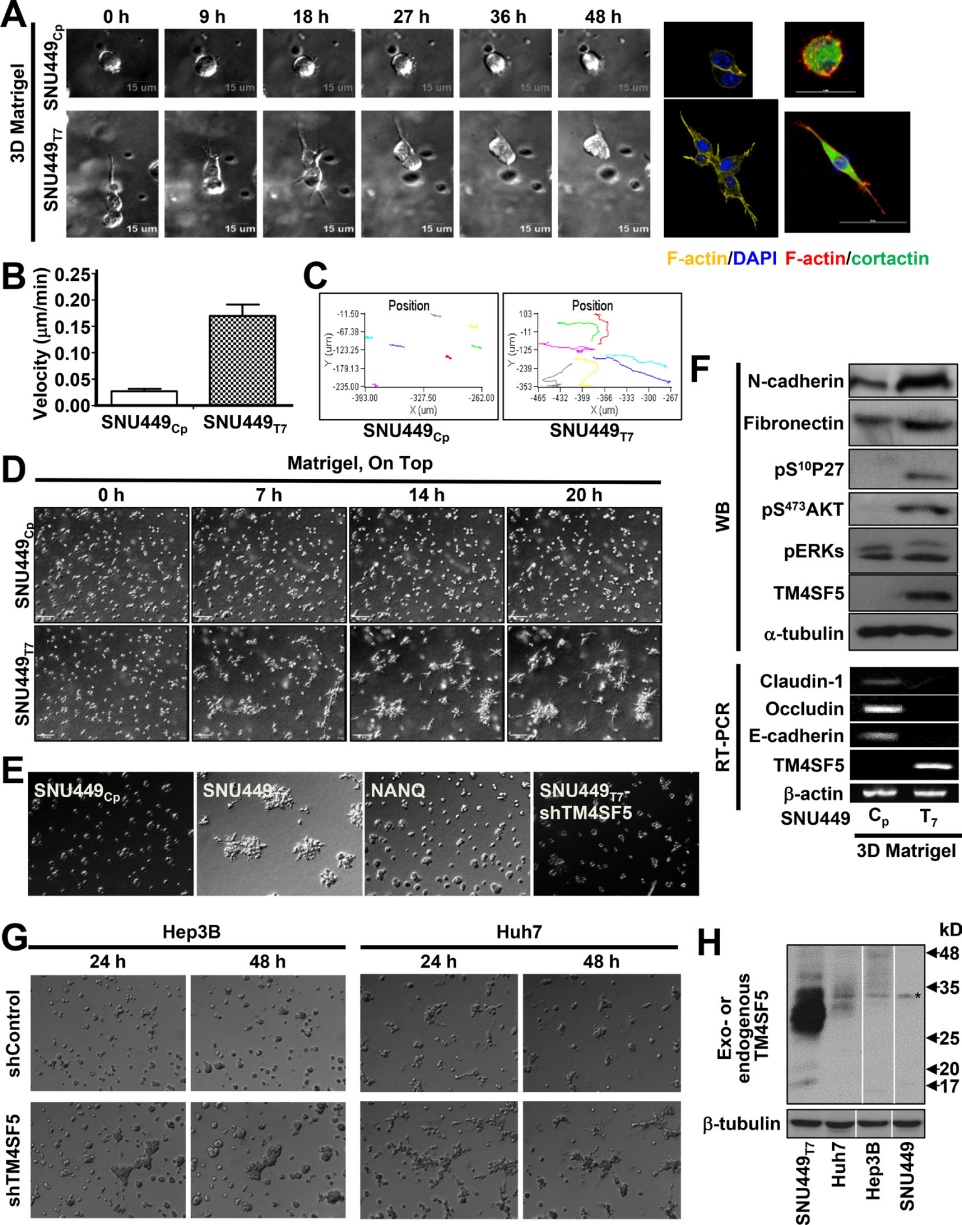
TM4SF5-expressing cells showed invasive behaviors in 3D Matrigel (**A**–**F**) Individualized cells stably infected with retroviral vector for Mock (SNU449_Cp_), TM4SF5 wildtype (SNU449_Tp_), or *N*-glycosylation mutant (N138A/N158Q, NANQ) or SNU449_Tp_ cells stably transfected with shRNA against TM4SF5 (shTM4SF5) were embedded in a 3D Matrigel (8~10 mg/ml), prior to imaging using a time-lapse microscopy with controlled CO_2_ (5%) and temperature (37°C) for 24 h or indicated times. The representative end point images were shown. Cells were individually imaged for their morphologies and then stained for DNA (DAPI), F-actin (YFP or Phalloidin), and cortactin (FITC) (A). Live cell images (*n* = 7) were used for cell tracking for graphic presentations at mean ± standard deviation (SD) (B and C). Individualized cells at higher density were live-imaged for 24 h and representatively-selected snap images at each indicated time were presented (D and E). See also Supplementary Movies 1 and 2. After live imaging for 20 h as in (D), cells were processed for RT-PCR or harvested for whole cell extracts, before standard immunoblottings using antibodies for the indicated molecules (F). (**G** and **H**) Hep3B and Huh7 hepatic cancer cells endogenously expressing TM4SF5 were embedded into 3D Matrigel, prior to live imaging, as above (G). Whole cell extracts of the cells were prepared and processed for the standard Western blot for TM4SF5 using rabbit polyclonal antibody against the C-terminus of TM4SF5. SNU449Tp cell extracts were a positive control. TM4SF5 could be N-glycosyated for multiple smear bands different in cell types. * depicts a nonspecific band. Data shown represent at least 3 independent experiments.

To examine the effects of TM4SF5 expression on cellular behavior, TM4SF5-negative (SNU449_Cp_) and positive (i.e., ectopically expressing TM4SF5; SNU449_Tp_) cells were embedded in 3D Matrigel and live imaged. Whereas individual SNU449_Cp_ cells maintained an amoeboid shape, TM4SF5-positive cells displayed mesenchymal shapes with protrusive tips and exhibited distal invasion over time (Figure 1A). SNU449_Tp_ cells had a greater velocity than SNU449_Cp_ cells (Figure 1B) and exhibited more persistent migration through the 3D Matrigel (Figure 1C).

Imaging of the embedded cells at a higher density revealed that the control SNU449_Cp_ cells did not exhibit any specific migratory patterns, whereas the SNU449_Tp_ cells gathered to form invasive foci following the aggressive migration of individual cells (Figure 1D and Supplementary Movies 1 and 2). Interestingly, cells expressing *N*-glycosylation-deficient TM4SF5 (TM4SF5-NANQ) or cells with suppression of TM4SF5 did not form invasive foci (Figure 1E), indicating that invasive foci formation depended on expression of wild type TM4SF5. Knowing that *N*-glycosylation-dependent localization of TM4SF5 to plasma membrane [11] was required for its function, it is likely that the function also depends on environmental factors outside of the SNU449_Tp_ cells, presumably via outside-to-inside signal transduction. Compared with SNU449_Cp_ cells, SNU449_Tp_ cells embedded in 3D Matrigel for 24 h showed increased mesenchymal markers, loss of epithelial markers, increased protein kinase B (Akt) activity, and increased cytosolic p27^Kip1^ level (i.e., pS^10^p27^Kip1^), which are downstream of TM4SF5 [12] (Supplementary Figure 1A and Figure 1F). In addition, endogenously TM4SF5-expressing Hep3B and Huh7 cells also showed TM4SF5 expression-dependent foci formation in 3D Matrigel (Figure 1G and 1H). Therefore, TM4SF5 promoted more persistent migration and invasive foci formation of cells embedded in 3D Matrigel.

### TM4SF5-mediated invasive foci formation in 3D Matrigel was regulated by TM4SF5-dependent intracellular signaling activity

**Figure 2:**
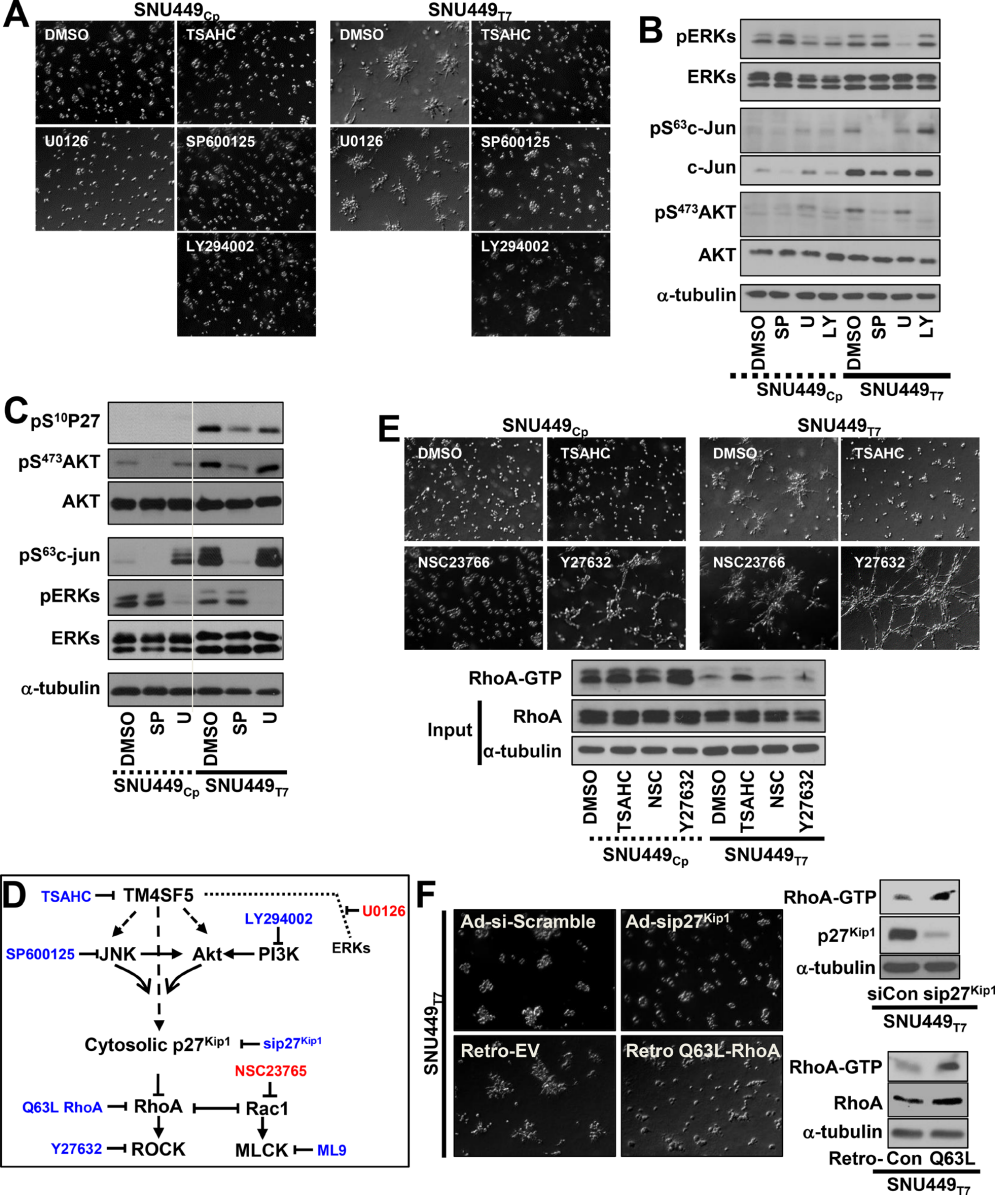
Invasive foci formation of cells in 3D Matrigel depended on TM4SF5-mediated intracellular signaling activities Cells were embedded into 3D Matrigel and manipulated, as explained in Figure 1 (**A**–**F**). In cases, diverse pharmacological inhibitors were included into the Matrigel during embedding, before live imaging for 17 h. The representative end point images were shown. Cells were then harvested for whole cell lysates, before standard Western blots for the indicated molecules (B and C). SP, U, and LY depict SP600123, U0126, and LY294002, respectively (A to C). Scheme of TM4SF5-mediated signaling network and relative inhibitors (D). In case, cells were treated with DMSO, TSAHC, NSC23766, or Y27632 during embedding, before live imaging or RhoA assay (E). In case, cells were infected with adenovirus (for scramble sequence or siRNA against p27^Kip1^, sip27^Kip1^) or retrovirus (for empty, EV, or constitutively active Q63L RhoA) for 24 h. See also Supplementary Movie3. The cell lysates were processed for the RhoA assay, as explained in a previous study [12] (F). Data shown represent 3 isolated experiments.

We next used pharmacological inhibitors to investigate whether TM4SF5-mediated signaling activities regulate foci formation. The anti-TM4SF5 reagent, 4′-(*p*-toluenesulfonylamide)-4-hydroxychalcone (TSAHC) [16] blocked TM4SF5-mediated foci formation. In addition, a Jun N-terminal kinase (JNK) inhibitor and phosphoinositide 3-kinase (PI3K) (and thereby Akt) inhibitor blocked foci formation by SNU449_Tp_ cells, with no effects on SNU449_Cp_ cells (Figure 2A). In contrast, an extracellular regulated kinase (ERK) inhibitor did not alter the cellular patterns of either SNU449_Cp_ or SNU449_Tp_ cells in 3D Matrigel, although the inhibitor abolished ERK activities as intended (Figure 2A and 2B). In consistent with previous reports [12, 17], the pharmacological inhibition of JNK or Akt in SNU449_Tp_ cells resulted in concomitant changes in total p27^Kip1^ and Ser10-phosphorylated p27^Kip1^ (i.e., pS^10^p27^Kip1^) levels (Figure 2C and Supplementary Figure 1). These changes led to the accumulation of pS^10^p27^Kip1^ in the cytosol, where p27^Kip1^ can bind and thereby inactivate RhoA [18]. Because TM4SF5 causes RhoA inactivation and the concomitant activation of Rac1 [12] (Figure 2D), inhibition of ROCK, which functions downstream of RhoA, resulted in invasive foci formation by SNU449_Cp_ cells in 3D Matrigel. In contrast, ROCK inhibition in SNU449_Tp_ cells resulted in a widespread network structure formation (Figure 2E and Supplementary Movie 3). Treatment with NSC23766 to inhibit Rac1 did not block the foci formation by SNU449_Tp_ cells or cause foci formation by SNU449_Cp_ cells (Figure 2E). This result suggests that TM4SF5-mediated invasive foci formation in 3D Matrigel was supported by the reduction in RhoA activity (Figure 2E, bottom). Furthermore, the suppression of p27^Kip1^, which was accomplished using an adenovirus containing shRNA against p27^Kip1^, and the retroviral-mediated introduction of constitutively-active (Q63L) RhoA abolished TM4SF5-mediated invasive foci formation in SNU449_Tp_ cells (Figure 2F).

Because RhoA is involved in cytoskeletal network organization and intracellular contractility [19], the roles of F-actin and the microtubule network in TM4SF5-mediated foci formation were examined. The inhibition of F-actin polymerization abolished invasive foci formation by SNU449_Tp_ cells and ROCK-inhibited SNU449_Cp_ cells in 3D Matrigel, and disruption of the microtubule network abolished the foci formation by SNU449_Tp_ cells (Supplementary Figure 2). Foci formation in ROCK-inhibited SNU449_Cp_ cells could result from the action (i.e., contractility, polarity, and/or traction force) and integrity of F-actin, rather than chemical-mediated side effects. Foci formation in ROCK-inhibited SNU449_Cp_ cells was in turn blocked by inhibition of extracellular MMP activities. TM4SF5 was indeed responsible for invasive foci formation by cells embedded in 3D Matrigel, which depended on intracellular signaling activity involving ROCK, cytoskeletal networks, and extracellular remodeling activities involving MMP2 activity.

### TM4SF5-mediated invasive foci formation in 3D Matrigel involved the remodeling of the extracellular environment

**Figure 3:**
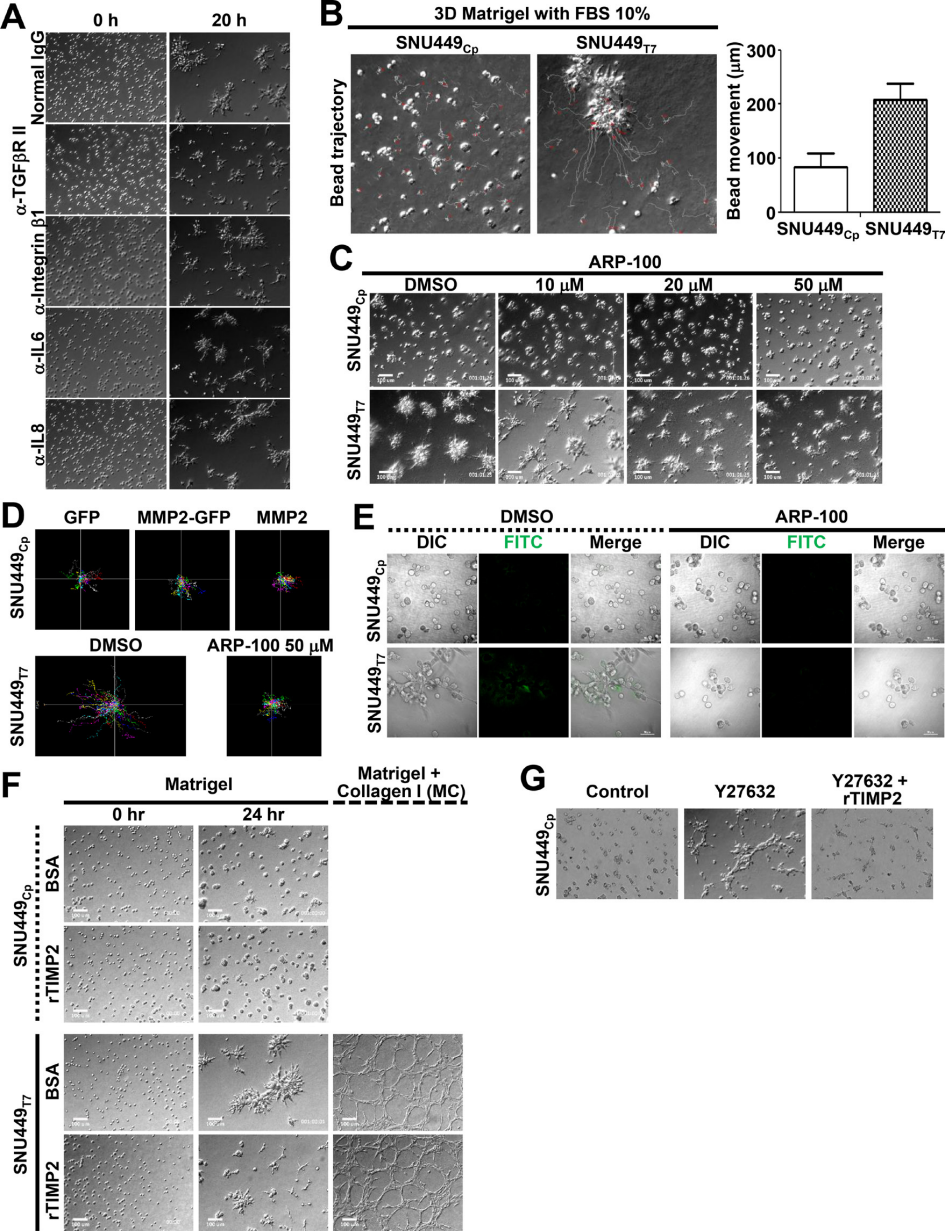
Extracellular matrix remodeling was important for the TM4SF5-mediated invasive foci formation in 3D Matrigel (**A**–**D**) Cells were embedded into 3D Matrigel, as above. During embedding, antibodies (A), beads (B), or diverse pharmacological inhibitors (C) were included into the Matrigel. In case, beads around the cells were tracked for their individual movements for a graphic presentation at mean ± SD (B). Then the cells were live imaged for 24 h. In case, cells stably transfected with GFP, MMP2-GFP, or MMP2 expression vectors were embedded and live-imaged for single cell tracking (*n* ≥ 10, D). (**E**) Cells were embedded into 3D Matrigel together with DQ-collagen to see ECM degradation by an appearance of green fluorescence upon its degradation. (**F**) Cells were embedded into 3D Matrigel or Matrigel and collagen I mixture (MC, 10 mg/ml : 2.5 mg/ml ratio) in the presence of control protein (BSA) or recombinant TIMP2 (rTIMP2, 200 ng/ml), and live-imaged for 24 h. Representative starting and end point snap images were shown. (**G**) Cells were embedded into 3D Matrigel in the presence of vehicle DMSO (Control), Y27632 (20 μM) alone, or Y27632 (20 μM) and rTIMP2 (200 ng/ml), and live-imaged for 24 h. Representative end point images were shown. Data shown represent 3 independent experiments.

We next explored the influence of extracellular cues on TM4SF5-dependent foci formation. TM4SF5-positive liver cancer cells show reduced expression of IL6 [15], and TM4SF5 is induced by TGFβ1-mediated signaling [20]. Integrin β1 is an adhesion receptor chain that binds the ECM and is known to collaborate with the tetraspanins [21]. We used antibodies to deplete IL6 or IL8 and to compete with and disrupt the ligand-binding functions of TGFβ receptor II (TGFβRII) or integrin β1 during the 3D embedding process to examine whether this might block TM4SF5-mediated foci formation. During 20 h of live imaging, treatment with anti-TGFβRII antibody partially blocked the invasive foci formation of SNU449_Tp_ cells but had no effect on SNU449_Cp_ cells. The other antibodies did not alter TM4SF5-dependent foci formation (Figure 3A). Although TGFβ1 treatment caused SNU449_Cp_ cells to slightly gather together, other reagents, including tumor necrosis factor α (TNFα), fibroblast growth factor (FGF), hepatocyte growth factor (HGF), platelet-derived growth factor (PDGF), phorbol 12-myristate 13-acetate (PMA), and IL6 did not induce foci formation (Supplementary Figure 3). Microbeads were also embedded and traced during the live imaging period. As time passed, the tracks of bead movements around the SNU449_Tp_ cells were changed to a greater extent (Figure 3B) than those around the SNU449_Cp_ cells. The direction of movement of the (non-proteolytic) beads was parallel to the migratory directions of the SNU449_Tp_ cells (Figure 3B), indicating that ECM remodeling occurred during cell movement. These observations suggest that TM4SF5-dependent invasive foci formation involved the massive deformation of the ECM network.

Our examination of the involvement of matrix metalloproteinases (*MMPs*) revealed that pharmacological inhibition of MMP2 abolished the foci formation by SNU449_Tp_ cells, whereas the inhibition of MMP9 did not. Neither treatment affected SNU449_Cp_ cells (Supplementary Figure 4A and data not shown). Treatment with the MMP2 inhibitor ARP-100 reduced invasive foci formation by SNU449_Tp_ cells in a dose-dependent manner, but it did not affect SNU449_Cp_ cells (Figure 3C). Although the overexpression of MMP2 in SNU449_Cp_ cells did not increase migration, the inhibition of MMP2 in SNU449_Tp_ cells decreased TM4SF5-mediated persistent migration in a 3D on-top system (Figure 3D). The inhibition of MMP2 did not affect TM4SF5 expression levels, as determined by immunoblotting with anti-TM4SF5 antibodies (Supplementary Figure 4B). Furthermore, the inhibition of MMP2 did not cause any changes in signaling activities downstream of TM4SF5 (Supplementary Figure 4C). ECM degradation visualized by dye-quenched (DQ) collagen fluorescence in SNU449_Tp_ cells, but not in SNU449_Cp_ cells, was blocked by treatment with the MMP2 inhibitor ARP-100 (Figure 3E). Treatment with recombinant tissue inhibitor of metalloproteinases (TIMP) 2 protein (rTIMP2) blocked TM4SF5-dependent invasive foci formation in 3D Matrigel (Figure 3F). Furthermore, the invasive foci formation in SNU449_Cp_ cells that was observed following ROCK inhibition could also be blocked by rTIMP2 treatment (Figure 3G). Therefore, in addition to intracellular signaling activities that presumably drive cytoskeletal network organization and migratory/invasive abilities, extracellular MMP2 activity might also be involved in deformation of the ECM during the TM4SF5-dependent invasive foci formation in 3D Matrigel.

### TM4SF5-positive cells in 3D gels consisting of both Matrigel and collagen I induced elongation to form networks

**Figure 4:**
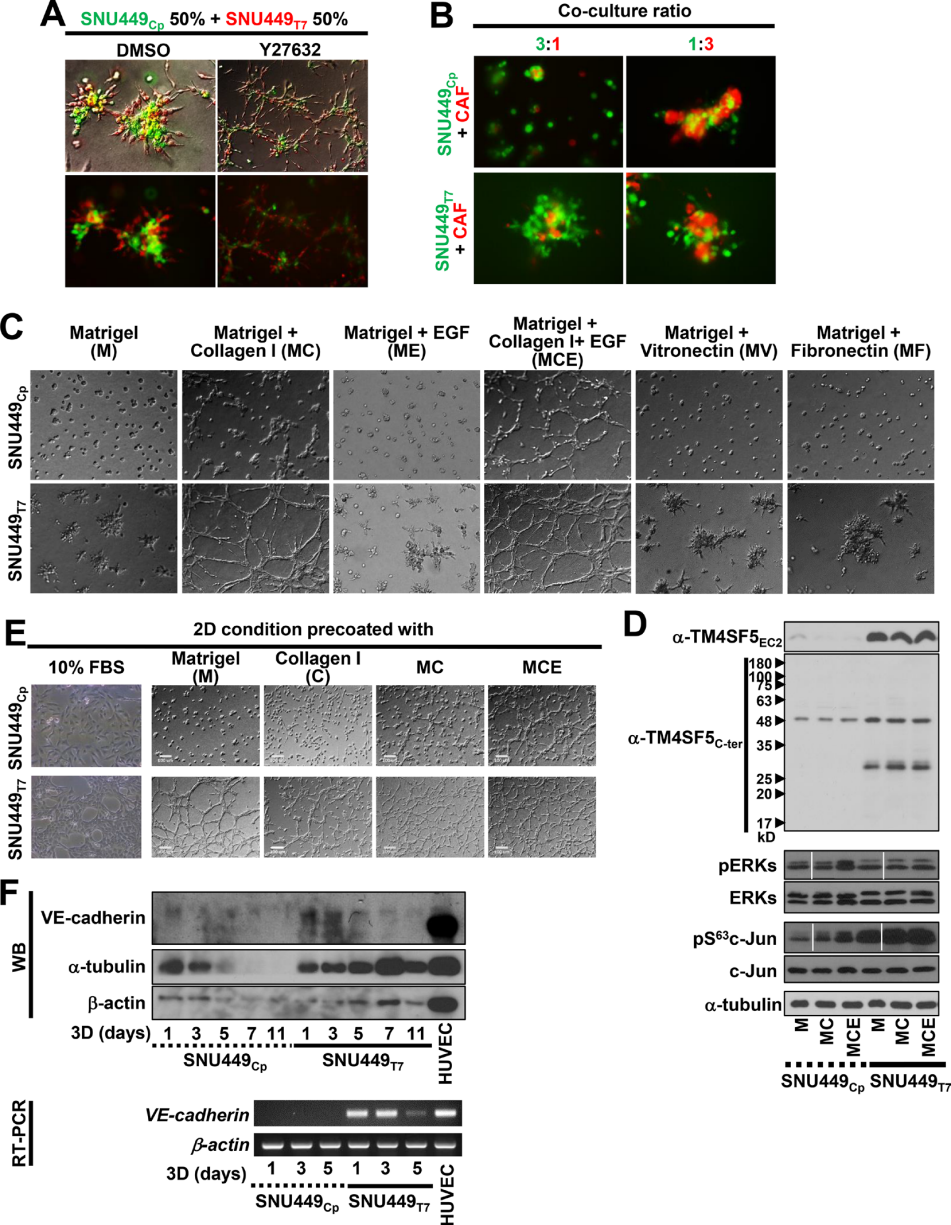
TM4SF5-null cells required more environmental factors to mimic TM4SF5-mediated formation of invasive foci and vasculogenic mimicry (**A**) SNU449_Cp_ and SNU449_Tp_ cells were labelled with CellTracker Green CMFDA and Red CMTPX, respectively, and then mixed at 50 to 50% ratio, before their 3D embedding with DMSO or Y27632 (20 μM) treatment. After 24 h incubation at a regular CO_2_ incubator, the cells were imaged. Location of SNU449_Tp_ cells outside of the cell clusters was statistically observed (15 out of 15 foci from 3 different experiments). See also Supplementary Movie 4. (**B**) SNU449_Cp_ cells labelled with CMFDA and cancer associated fibroblasts (CAFs) labelled with CMTPX were mixed at 3 to 1 or 1 to 3 ratio, respectively, prior to embedding into 3D Matrigel. Then image was saved after an incubation for 24 h in a CO_2_ incubator. (**C** and **D**) Cells were embedded into 3D Matrigel alone (M) or together with either collagen I (MC), vitronectin (MV), or fibronectin (MF) without or with EGF (MCE), before live imaging for 24 h. The representative end point images were shown (C). See also Supplementary Movies 5 and S6. Alternatively, the cells were harvested for whole cell lysates and processed for immunoblottings (D). (**E**) Cells were seeded onto 2D flat thin culture condition precoated with 10% FBS-containing media (10% FBS), Matrigel (M), Collagen I (C), or Matrigel together with collagen I (MC, 20 μl of 10 mg/ml ECM solution/0.7 cm^2^) without or with EGF (50 ng/ml) treatment, before live imaging for 24 h. (**F**) Cells embedded in 3D Matrigel for the indicated times were processed for RT-PCR or harvested for the whole cell extracts, before standard Western blots for VE-cadherin and β-actin, and HUVEC cells were used for a positive control. Data shown represent 3 independent experiments.

We next tested whether different neighboring cells might affect TM4SF5-mediated foci formation. First, we mixed SNU449_Cp_ and SNU449_Tp_ cells in 3D Matrigel. Interestingly, the SNU449_Tp_ cells localized outside of the cell clusters consisting of the both cell types (Figure 4A and Supplementary Movie 4), indicating that TM4SF5-positive SNU449_Tp_ cells had a greater invasive capacity via outreaches from each foci. A ROCK inhibitor was applied to the co-culture of SNU449_Cp_ and SNU449_Tp_ cells to determine how they behaved. Interestingly, upon co-culture in the presence of the ROCK inhibitor, the cells formed network-like tube patterns, with more SNU449_Tp_ cells at the linking areas (Figure 4A, rights). Co-culture of SNU449_Cp_ or SNU449_Tp_ cells with cancer-associated fibroblasts (CAFs) in 3D Matrigel at different ratios (1:3 or 3:1, respectively) showed that SNU449_Cp_ cells formed compact clusters that were surrounded by CAFs and SNU449_Tp_ cells located outside of the CAFs (Figure 4B). Therefore, the presence of SNU449_Tp_ cells or CAFs could be enough to induce SNU449_Cp_ cell clustering. Because SNU449_Tp_ cells are migratory more than SNU449_Cp_ cells [13] and because CAFs can produce collagen I, we speculated that the addition of collagen I to 3D Matrigel might affect the cellular behaviors described above. The addition of collagen I to 3D Matrigel (i.e., MC condition) resulted in a slight foci formation by SNU449_Cp_ cells. The SNU449_Tp_ cells formed network-like structures in 3D Matrigel (Figure 4C and Supplementary Movie 5), in a manner similar to human umbilical vein endothelial cell (HUVEC)-mediated tube formation on Matrigel [22]. However, unlike collagen I, the addition of vitronectin or fibronectin to 3D Matrigel did not cause SNU449_Cp_ cells to form invasive foci (Figure 4C). Furthermore, the addition of EGF to the invasive foci of SNU449_Cp_ cells formed in the MC condition resulted in an endothelial-like network that resembled that of SNU449_Tp_ cells in the MC condition without EGF (Figure 4C and Supplementary Movie 6). Meanwhile, the treatment with EGF or addition of either vitronectin or fibronectin to SNU449_Tp_ cells forming invasive foci in 3D Matrigel (i.e., ME, MV, or MF condition, respectively) did not cause the endothelial-like networks (Figure 4C), indicating that specific cues underlie these networks. Therefore, TM4SF5-positive epithelial cancer cells might form and even mimic vasculogenic networks, suggesting that their aggressive metastatic potential depends on TM4SF5 expression and specific extracellular cues. However, TM4SF5 expression levels were not enhanced in SNU449_Cp_ cells under any of these 3D conditions (M, MC, or MCE), indicating that the activity of signaling molecules downstream of TM4SF5 might be facilitated by the additional component(s) outside of the cells (Figure 4D).

Treatment of rTIMP2 did not block the endothelial-like network formation of SNU449_Tp_ cells under the MC condition, although it did block formation of foci (Figure 3F). It may thus be likely that whereas foci formation depending on both intracellular signaling activity and extracellular remodeling was blocked by rTIMP2 treatment (Figure 3G), network-like structure formation depending on additional collagen I-dependent signaling in 3D Matrigel was not blocked by rTIMP2 treatment (Figure 3F). To determine whether the endothelial-like network formation of SNU449_Tp_ cells was dependent on ECM (i.e., Matrigel and collagen I together)-mediated intracellular processes, cells on Matrigel-coated 2D surfaces were imaged. Unlike SNU449_Cp_ cells, SNU449_Tp_ cells displayed endothelial-like networks under the 2D condition (Figure 4E). These networks were similar to those seen in HUVECs cultured on 2D Matrigel. Furthermore, SNU449_Tp_ cells cultured in the 3D MC condition expressed VE-cadherin (Figure 4F), indicating that their vasculogenic cell transformation properties might be transcriptionally induced under 3D MC conditions and that both intracellular biochemical processes such as VE-cadherin expression and extracellular cues (i.e., additional collagen I) were involved in the endothelial-like network formation.

### TM4SF5-positive cells in 3D collagen I gels more dramatically disseminated from spheroids with more invadopodia formation

**Figure 5:**
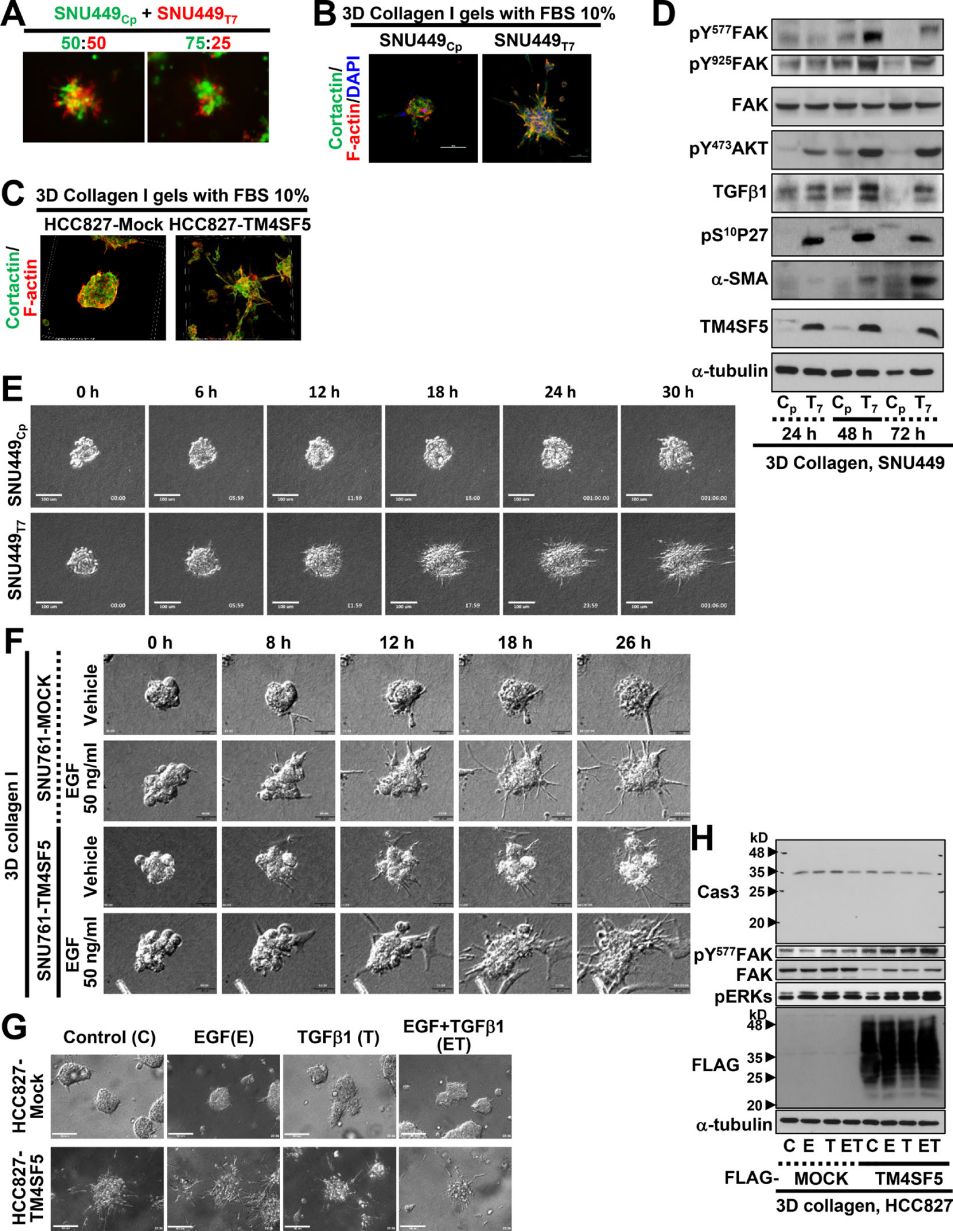
TM4SF5-expressing spheroids in 3D collagen I gel showed invasive outgrowth with greater invadopodia formation (**A**) Individualized SNU449_Cp_ and SNU449_Tp_ cells labelled with Green CMFDA and Red CMTPX, respectively, were embedded at the indicated ratio in 3D collagen I (2.5 mg/ml) gels. After 24 h incubation in a CO_2_ incubator, the cells were imaged. (**B** and **C**) Spheroids from the SNU449_Cp_ or SNU449_Tp_ cells (B) or HCC827-Mock or HCC827-TM4SF5 cells (C) were prepared using hanging drop systems, and spheroids at a similar size were embedded in 3D collagen I gels. After 24 h incubation at a CO_2_ incubator, cells were then stained for nuclei (DAPI), F-actin (phalloidin), and cortactin (FITC). (**D**) Spheroids from the SNU449_Cp_ (C_p_) or SNU449_Tp_ (T_7_) cells were manipulated for spheroid embedding into 3D collagen I gels, as explained above. The cells were incubated at a CO_2_ incubator for 24, 48, or 72 h, prior to harvests for whole cell lysates and standard Western blots for the indicated molecules. (**E**) SNU449 spheroids in 3D collagen I gels were live imaged for 30 h, and representative images were shown for the indicated times. See also Supplementary Movies 7 and 8. (**F**) Spheroids from SNU761-Mock or SNU761-TM4SF5 cells were embedded into 3D collagen I gels with vehicle or EGF (50 ng/ml) and then live imaged for 30 h. Representative images for each indicated time were shown. (**G** and **H**) Spheroids from HCC827-Mock or HCC827-TM4SF5 cells were embedded in 3D collagen I gels without (Control) or with EGF (E) or TGFβ1 (T) alone or the both (ET) treatment. Then cells were live imaged for 24 h, and the end point snap images for each condition were shown. Whole cell lysates were then prepared, before immunoblottings. Data shown represent 3 independent experiments.

In addition to the co-culture of SNU449_Cp_ and SNU449_Tp_ cells at a 1:1 ratio, co-culture at a 3:1 ratio still resulted in SNU449_Tp_ cells at the outward edges of the cell clusters (Figure 5A). In addition, staining for F-actin and cortactin, which are indicative of invadopodia, revealed that SNU449_Tp_ cells, but not SNU449_Cp_ cells, in 3D collagen I gels displayed dramatic invadopodia formation with aggressively invasive outgrowth (Figure 5B). A non-small cell lung cancer (NSCLC) line, HCC827-TM4SF5, also showed aggressive invasion with greater invadopodia formations than HCC827-Mock cells (Figure 5C). The greater invadopodia formation of TM4SF5-positive cells correlated with enhanced phosphorylation of FAK, Akt, and p27^Kip1^, and increased expression of α-smooth muscle actin (α-SMA), all of which function downstream of TM4SF5 for a pro-migratory capacity (Figure 5D and Supplementary Figure 1A).

When SNU449_Cp_ and SNU449_Tp_ spheroids were embedded in a 3D collagen I gel system and live imaged for 30 h, the SNU449_Tp_ cells showed greater growth and invasive outward protrusions than the generally static SNU449_Cp_ cells (Figure 5E and Supplementary Movies 7 and 8). Furthermore, the spheroids from two different SNU449_Tp_ and SNU449_Tp_ clonal cell lines embedded in 3D Matrigel (with 10% FBS-containing media) also showed more outward protrusions, even with additional treatment with EGF or TGFβ1 (Supplementary Figure 5). SNU761-TM4SF5 and HCC827-TM4SF5 cells also showed aggressive outgrowth under basal and additional EGF-treatment conditions, compared with control SNU761-Mock and HCC827-Mock cells, respectively (Figure 5F and 5G). Additional treatment with EGF, TGFβ1, or both enhanced the invasive outgrowth of TM4SF5-expressing cells to a greater extent than that of cells lacking TM4SF5 (Figure 5F and 5G). The enhanced outgrowth of TM4SF5-expressing cells was associated with TM4SF5-mediated FAK activity, but caspase activation was not observed, indicating that there was no caspase-dependent apoptosis even after a long culture period under 3D gel conditions (Figure 5H).

## DISCUSSION

**Figure 6:**
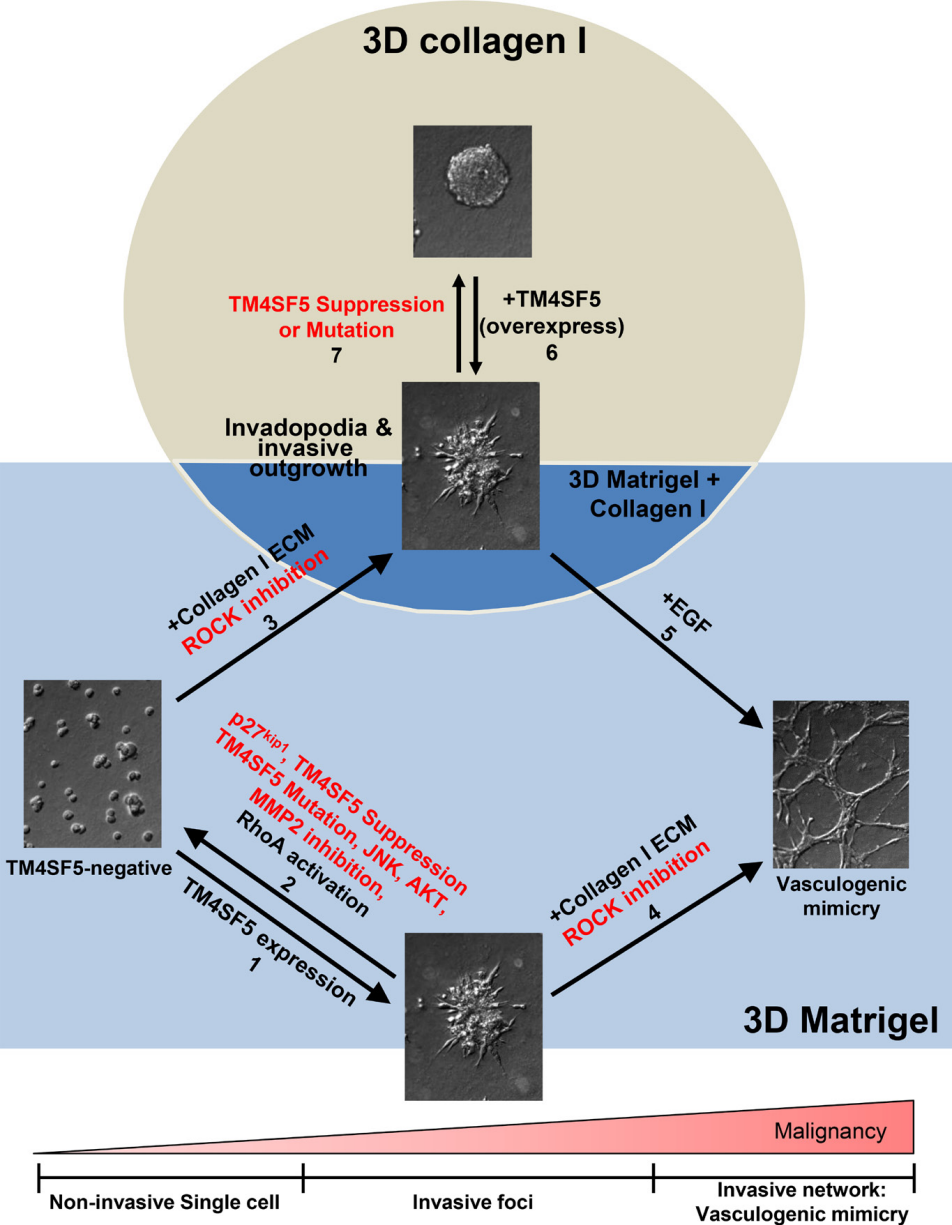
TM4SF5-expressing cells showed greater metastatic properties in 3D ECM gels even with requirements of environmental factors fewer than TM4SF5-lacking cells In 3D Matrigel system, TM4SF5-expressing cells formed invasive foci, whereas control TM4SF5-lacking cells stayed steady (arrow 1). However, the TM4SF5-dependent invasive foci was abolished by suppression or mutation of TM4SF5 or inhibition or blocking of TM4SF5-mediated signaling activities (arrow 2). However, invasive foci formation by TM4SF5-lacking cells was possible upon being embedded in the gels consisting of Matrigel and collagen I as well (arrow 3). Meanwhile, TM4SF5-expressing cells formed vessel-like tube networks (i.e., vasculogenic mimicry-like), when they were embedded in gels of Matrigel and collagen I (arrow 4). For TM4SF5-lacking cells to form vasculogenic mimicry, they should be embedded in the gels consisting of Matrigel and collagen I additionally in the presence of EGF treatment (arrow 5). Meanwhile, spheroids from TM4SF5-expressing cells in 3D collagen I gels showed aggressive outgrowth and invadopodia formation (arrow 6), which was abolished by suppression or mutation at *N*-glycosylation residues of TM4SF5 (arrow 7).

This study demonstrates that TM4SF5-expressing cells can efficiently utilize environmental cues, such as ECM components and soluble factors EGF and TGFβ1, to achieve greater metastatic potentials than cells lacking TM4SF5. TM4SF5-expressing cells formed invasive foci in 3D Matrigel with 10% serum and also displayed endothelial-like networks, presumably vasculogenic mimicry, upon an addition of collagen I to the 3D Matrigel. In addition, TM4SF5-expressing spheroids showed more aggressive invadopodia formation and invasive outgrowth in 3D collagen I gels. These TM4SF5-mediated effects were blocked by modulation of the signaling activities downstream of TM4SF5. Furthermore, cells or spheroids without TM4SF5 expression showed no or less effective phenotypes, and only demonstrated TM4SF5-dependent phenotypes upon treatment with additional environmental cues (Figure 6).

TM4SF5-expressing epithelial cancer cells appeared to have angiogenic or vasculogenic properties; for examples, under 2D conditions, they formed a network-like growth pattern, and in 3D gels made of Matrigel and collagen I, they displayed endothelial-like networks with VE-cadherin mRNA and protein expression. Vasculogenic mimicry is clinically well-known to indicate its high metastatic potential [23–25]. The angiogenic and vasculogenic transformation mechanisms of TM4SF5-positive cancer cells need further exploration. TM4SF5-mediated cellular or spheroidal behaviors that duplicate many of the sophisticated aspects of metastatic processes were regulated by both intracellular signaling activity and the action of extracellular cues. Such TM4SF5-dependent phenotypes in *in vivo*-like 3D gel systems could be promising platforms to explore the mechanistic aspects of TM4SF5-dependent cancer metastasis and to screen for anti-metastatic reagents.

Here we observed that TM4SF5 expression facilitated the formation of invasive foci in a 3D Matrigel environment, whereas this effect was not observed in TM4SF5-lacking cells. We found that the co-culture of CAFs with TM4SF5-negative cells resulted in the surrounding of SNU449_Cp_ cells by CAFs. Thus, it may not be ruled out that CAFs may alter the ECM topology to allow the clustering of TM4SF5-negative cells together with CAFs. Furthermore, it may be likely that TM4SF5 expression itself appears to bypass, at least partially, the role of CAFs. Although *in vivo* tumor lesions would be surrounded by complicated soluble factors, ECM, and neighboring cells, TM4SF5-expressing cells may have a greater potential to survive and metastasize even in the presence of fewer complicated environmental cues. To this point, TM4SF5-positive cells formed foci in 3D Matrigel, whereas TM4SF5-negative cells needed additional collagen I. Furthermore, TM4SF5-positive cells showed endothelial-like network structures in 3D Matrigel and collagen I gels, whereas TM4SF5-negative cells formed such network structures in the same 3D gels only when EGF was further. While acquiring these functions, TM4SF5-positive cancer cells may remodel environments to be more favorable for their metastasis than TM4SF5-negative cells. It is also likely that TM4SF5 promotes the synthesis and secretion of environmental cues via more efficient intracellular signaling or communication with neighboring cells, eventually leading to the requirement for fewer extracellular cues to achieve greater metastatic behaviors. Consistent with this idea is the observation that TM4SF5-expressing cells induce more VEGF to trigger the angiogenic activities of neighboring endothelial cells than do TM4SF5-negative cells [22]. TM4SF5-mediated invasions on gelatin-precoated culture dishes or transwell systems involve EGFR activation even without EGF treatment, indicating activation of ligand-independent c-Src/EGFR [14]. In addition, TM4SF5 expression results in the activation of FAK and STAT3, even without ECM-adhesion stimulation [12] and IL6 treatment [15], respectively. Here, TM4SF5-positive cancer cells expressed VE-cadherin and exhibited elongations to form networks, as if they were endothelial cells. Therefore, TM4SF5 reduced the requirement for extracellular components for the activation of enhanced intracellular signaling and cellular functions.

In addition to TM4SF5-mediated intracellular signaling activities, the extracellular cues in the 3D ECM gel system were shown to be important for the TM4SF5-mediated metastatic behaviors. While TM4SF5-positive cells, but not TM4SF5-negative cells, in 3D Matrigel mediated invasive foci formation, the extracellular environments were reformed as visualized by the movement of beads along or the degradation of collagen I around TM4SF5-positive cells. Furthermore, blocking TGFβRII with an antibody or inhibiting MMP2 with a pharmacological inhibitor or recombinant TIMP2 protein abolished invasive foci formation. Thus, it is likely that extracellular cues, including a multifunctional cytokine TGFβ1 and MMP2, may be involved in expression of TM4SF5 [20] and foci formation. Interestingly, MMP2 inhibition blocked the foci formation but did not block TM4SF5-mediated intracellular signaling activity or avidity, which are known to involve FAK, Akt, STAT5, c-Jun, p27^Kip1^, and phospho-p27^Kip1^ [21]. This observation indicates that both aspects of intracellular signaling activities and extracellular environmental dynamics promote TM4SF5-mediated invasive foci formation and metastatic behaviors. TM4SF5-expressing cells increased p130Cas phosphorylation in cells under normal 2D normal culture conditions, but the cells cultured in 2D in the presence of different concentrations of collagen I (5–50 μg/ml) showed adhesion-dependent p130Cas phosphorylation without additional TM4SF5-dependency (data not shown). TM4SF5 could thus be a promising target for the development of anti-metastatic reagents.

## MATERIALS AND METHODS

### Cells

Parental SNU449, SNU761 hepatocellular carcinoma, or HCC827 lung cancer cells lacking TM4SF5, or Hep3B or Huh7 hepatocellular carcinoma endogenously expressing TM4SF5 (Korean Cell Bank) were confirmed for their identities by the provider, and either infected with control retrovirus (SNU449_Cp_) or TM4SF5-retrovirus (SNU449_Tp_) as previously described [12], or transiently or stably transfected with a mock control or a FLAG-tagged TM4SF5 plasmid [13]. Stable cells were selected with 500 μg/ml G418 (A.G. Scientifics). The cells were maintained in RPMI-1640 (WelGene Inc., Korea) containing 10% fetal bovine serum (FBS, GenDEPOT Inc., Barker, TX) and 1% penicillin/streptomycin (GenDEPOT Inc.). Cancer-associated fibroblasts (CAFs) were a kind gift from Prof. Young-Joon Surh (Seoul National University). In certain experiments, the cells were transfected with the indicated expression control vector, TM4SF5 shRNA (shTM4SF5) [26], or TM4SF5 *N*-glycosylation mutants [N138A/N155Q (NANQ)] [15]. Alternatively, the cells were infected with control adenovirus or adenovirus expressing siRNA against p27^Kip1^ [27], or they were infected with retrovirus containing a constitutively active mutant RhoA (Q63L). In cases, different cells were co-cultured in 3D gel conditions at the indicated mixture ratio.

### Antibodies and reagents

Antibodies and reagents used in this study include 10× RPMI, anti-α-tubulin (Cat. # T5168), anti-α-SMA (Cat. # A2547), cytochalasin D, paclitaxel, Phorbol 12-myristate 13-acetate (PMA), polystyrene micro-particles (3 μm) (Sigma-Aldrich), anti-p27^Kip1^ (Cat. #; 610241), anti-pY^397^FAK (Cat. #; 611723), anti-paxillin (Cat. #; 610569), anti-cortactin (Cat. #; 610050), anti-N-cadherin (Cat. #; 610921) (BD Biosciences), the MMP2 and MMP9 inhibitors (Calbiochem), Matrigel™ Basement Membrane Matrix, Rat tail collagen type I (#354236, Corning), anti-pS10p27^Kip1^ (Cat. #; AP31919, Abgent), anti-pS^63^c-Jun (#9261), anti-c-Jun (#9165), anti-cofilin (#3312), anti-AKTs (#9272), anti-FLAG (#2368), anti-pERKs (#9101), anti-ERKs (#9102), anti-pY^694^STAT5 (#9314), anti-pY^118^paxillin (#2541), anti-caspase-3 (#9662), anti-VE-cadherin (#2158) (Cell Signal Technology), anti-integrin α2 (Cat. #; 09791D, Pharmingen), anti-integrin β1, (Cat. #; MAB1987Z, Millipore), ARP-100, anti-pS^473^AKT (Cat. #; SC-7985), anti-E-cadherin (Cat. #; SC-8426), anti-FAK (Cat. #; SC-558), anti-pY^861^FAK (Cat. #; SC-16663), anti-pY^925^FAK (Cat. #; SC-11766), anti-STAT5 (Cat. #; SC-835), anti-TGFβRII (Cat. #; SC-220), anti-IL-6 (Cat. #; 130326), anti-IL-8 (Cat. #; 376750) (Santa Cruz Biotechnology, Inc.), anti-pY^577^FAK (Cat. #; 44614G), diaminophenylindole (DAPI), rhodamine phalloidin (Invitrogen), anti-RhoA (Cat. #; R73920, Transduction lab), CellTracker™ Green CMFDA dye, CellTracker™ Red CMPTX dye, HRP-conjugated secondary antibodies (Thermo Scientific), anti-Fibronectin (Cat. #; A0245, Dako), SP600125 (a specific JNK inhibitor), U0126 (a specific MEK inhibitor that leads to ERKs inhibition), LY294002 (a specific PI3K inhibitor that leads to Akt inhibition also), Y27632 (a specific ROCK inhibitor), NSC23766 (a specific RAC1 inhibitor), paclitaxel (a promoter of microtubule assembly to block its depolymerization) (LC Labs), ML9 (a specific MLCK inhibitor), cytochalasin D (a specific inhibitor of actin polymerization) (Calbiochem), EGF, FGF, HGF, IL-6, PDGF, TGFβ1, and TNF-α (PeproTech). The antibodies against TM4SF5 were made in-house using extracellular domain 2 (EC2, anti-TM4SF5_EC2_) [12] or using an intracellular C-terminal sequence peptide (anti-TM4SF5_C-ter_) at a 1:10,000 dilution. The primary antibodies were used at a 1:1000 ratio with Tris-buffered saline-0.05% Tween solution and the proper anti-mouse or anti-rabbit IgG secondary antibody were used at a 1:5000 ratio.

### Western blotting

Whole cell lysates from 2D cultures were prepared, as described previously [28].

Cells embedded in 3D ECM were transferred into ice-cold 1.7 ml tubes and centrifuged at 2,500 × *g* for 1 min to remove any residual medium. The cell pellets within the ECM masses were washed with ice-cold PBS (3 times on ice) and then homogenized in a modified RIPA buffer (50 mM Tris-HCl, pH 7.4, 150 mM NaCl, 1% NP-40, and 0.25% sodium deoxycholate) with 0.1% SDS, 1 mM Sodium orthovanadate and protease inhibitor cocktail (GenDepot Inc., Barker, TX) by repeated pipetting using truncated pipette tips. The extracts were centrifuged at 12,000 × *g* for 10 min at 4°C. The levels of α-tubulin were used to normalize the samples for equal protein loadings in standard immunoblots.

### RT-PCR

Total RNA was isolated using TRIzol Reagent (Invitrogen), and complementary DNA (cDNA) was synthesized using amfiRivert Platinum cDNA synthesis master mix (GenDEPOT Inc.) according to the manufacturer's instructions. The cDNA was subjected to RT-PCR using Dream Taq Green PCR master mix (Thermo Scientific, San Jose, CA). The primer sequences were as follows: β-actin forward: 5′-TGA CGG GGT CAC CCA CAC TGT GCC CAT CTA-3′ and reverse: 5′- CTA GAA GCA TTT GCG GTG GAC GGA GGG-3′, claudin-1 forward: 5′-TGA GGA TGG CTG TCA TTG GG-3′ and reverse: 5′-AAA GTA GGG CAC CTC CCA GA-3′, VE-cadherin forward: 5′-AGG CAT AGC ATT GGA TAC TC-3′ and reverse: 5′-CTC GCA GAA GGT GAA CTC-3′, occludin forward: 5′-ACA GAC TAC ACA ACT GGC GG-3′ and reverse 5′-TCA CAG AGG TTT GGC TTC CG-3′, CDH1 (E-Cadherin) forward: 5′-TGC CCA GAA AAT GAA AAA GG-3′ and reverse: 5′-GTG TAT GTG GCA ATG CGT TC-3′, and TM4SF5 forward: 5′-CTG CCT CGT CTG CAT TGT GG-3′ and reverse: 5′- CAG AAG ACA CCA CTG GTC GCG-3′.

### Cell culture in 3D ECM gels and time-lapse imaging

For 3D on-top model, fifty microliters of thawed Matrigel (10 mg/ml, Corning) was laid on to a Lab-Tek 8-well chamber (Cat. #: 154534, 0.7 cm^2^ bottom area/well, Nunc) and allowed to solidify by incubating it for 30 min at 37°C. A cell suspension containing 50,000 cells within a fresh media containing 10% of ECM gel (500 μl, 1 mg/ml) was overlaid on to the bottom gel and incubated for another 30 min at 37°C in order for the cells to be surrounded by the matrix. Three-dimensional (3D) collagen type I gel model was also prepared using rat tail collagen type I (Corning). Collagen I mixtures (2.5 mg/ml) were prepared by adding the appropriate volumes of 10× reconstitution buffer (260 mM sodium bicarbonate and 200 mM HEPES) and 10× RPMI (Sigma Aldrich). To adjust the pH of the collagen solution to 7.2–7.4, an ice-cold solution of 2 N NaOH was used. The neutralized collagen solution was incubated on ice for 3-5 min to allow the pH to equilibrate, and then the solution was centrifuged at 10,000 × *g* for 3 min at 4°C to eliminate air bubbles. A mixture of Matrigel and either additional collagen I, vitronectin, or fibronectin gel (at a ratio of 5 mg/ml and 2.5 mg/ml, respectively) was also prepared using the same procedure described above, except that the additional ECM was prepared in a concentration of 2× and mixed with Matrigel at a 1:1 ratio. In some cases, the indicated concentration of vitronectin (Advanced BioMatrix) or fibronectin (BD Biosciences) was added to the Matrigel. For the co-culture experiments, different cell lines were stained separately with distinct CellTrackers™ (Green CMFDA or Red CMPTX, Thermo Scientific, San Jose, CA) in advance, according to the manufacturer's instructions. Inhibitors, cytokines, or neutralizing antibodies were added to the 10% ECM-containing upper layer. Recombinant TIMP2 (rTIMP2), was mixed together with the bottom layer of Matrigel. Time-lapse DIC cell images were captured with an IX81-ZDC (Olympus, Japan) for 24 h at 37°C. The microscope was equipped with a Chamlide Incubator System (Live Cell Instrument, Korea), and the environmental chamber mounted on the microscope was constantly maintained at 37°C, 5% CO_2_, and 95% humidity.

### Imaging of cells on flat (thin) 2D culture condition

Cells were seeded onto ECM-precoated wells (20 μl,, 10 mg/ml/0.7 cm^2^) for 24 h, before imaging their cellular network phenotypes.

### 3D immunofluorescence analysis

Cells were cultured within polydimethylsiloxane prepolymer (PDMS) glass coverslips [28], and fixed directly with 4% formaldehyde for 30 min at room temperature (RT), and subsequently treated with 100 mM glycine to quench any residual aldehyde groups. After washing with PBS, the cells were permeabilized with 0.5% Triton X-100 for 30 min at RT and blocked for 2 h with PBS containing 3% BSA. In some cases, the cells were stained with either fluorescein-labeled phalloidin (Molecular Probes, 1:250) or Alexa Fluor^®^ 488-labeled anti-cortactin (Millipore, 1:200) at 4°C overnight. The cells were then washed with wash buffer (130 mM NaCl, 13 mM Na_2_HPO_4_, and 3.5 mM NaH_2_PO_4_, pH 7.4). The nuclei were counterstained with DAPI. Images were captured at 37°C using a confocal microscope with a Nikon Plan Apochromat 60×/1.4 N.A. oil objective (Nikon Eclipse Ti; Nikon, Japan) and analyzed using the NIS software (Nikon) or IMARIS imaging software (Bitplane AG, Zurich, Swiss). The confocal z-stack images obtained from NIS software were reconstructed into 3D images with the aid of the IMARIS software, Easy 3D mode. The visualization analysis of select images was performed using the ImarisColoc and Surpass module.

### Cell motility and ECM deformation assay

Cell motility, tracking, and ECM deformation were measured after the collection of sequential time-lapse images. For the ECM deformation assay, polystyrene-based micro-particles (microbeads, 3μm, Sigma) were overlaid onto the ECM together with the cells. Sequential DIC images were analyzed with MetaMorph software (Molecular Devices, Sunnyvale, CA) using the automatic Track Objects feature with the search box surrounding the entire cell or the microbead. Manual adjustments were made where the software could not identify the correct trajectory of the cells or beads. The velocities and trajectories of the cells or beads were graphed using the built-in feature.

### ECM degradation assay using DQ^™^-collagen IV

Three-dimensional ECM (40 μl) consisting of Matrigel (10 mg/ml) and DQ™-collagen type IV (2.5 mg/ml, Life Technologies) at a volume ratio of 10:1 was overlaid onto a Lab-Tek 8 chamber borosilicate cover glass system. After a 30 min incubation at 37°C, SNU449_Cp_ or SNU449_Tp_ cells (35,000 cells/condition) were overlaid onto the ECM. After another 30 min incubation at 37°C, each group was overlaid with 300 μl of complete medium containing 10% Matrigel and either DMSO (Control) or ARP-100 (50 μM). The cells were then incubated for 20-24 h, and DIC and fluorescence images were randomly obtained using the Nikon Plan Apochromat 40×/0.95 N.A. objective of a Nikon Eclipse Ti microscope (Nikon, Japan).

### Spheroid formation and invasive outgrowth assay

Cells with modulated TM4SF5 expression levels were processed for spheroid formation using Perfecta3D^®^ 96-well hanging-drop plates (3D Biomatrix, Ann Arbor, MI). The spheroids were collected by low-speed centrifugation and embedded into 3D collagen I gels, as reported previously [28]. Time-lapse images were collected for the indicated periods using an IX81-ZDC microscope (Olympus, Japan) as described above.

### RhoA assay

Cell lysates were prepared from the indicated conditions in 3D Matrigel condition with diverse pharmacological inhibitors treatments and proceeded to RhoA assay via analyzing RhoA-GTP levels, as previously explained [12].

### Statistical methods

Student's *t*-tests were performed for comparisons of mean values to determine significance. *p* values < 0.05 were considered significant.

## SUPPLEMENTARY MATERIALS FIGURES AND VIDEOS



















## Abbreviations

2D or 3D: 2 dimensional or 3 dimensional
CAFs: cancer-associated fibroblasts
EC2: extracellular loop 2
ECM: extracellular matrix
DAPI: diaminophenylindole
HRP: horseradish peroxidase
FAK: focal adhesion kinase
NSCLC: Non-small cell lung cancer
MMP2: matrix metalloproteinase 2
ROCK: *Rho*-associated protein kinase
rTIMP2: recombinant tissue inhibitor of metalloproteinase 2
PDMS: polydimethylsiloxane prepolymer
SNU449Cp: A stable cell clone pooled from control vector-infected SNU449 cells
SNU449Tp: A stable cell clone of SNU449 cells infected with retroviral vector inserted with TM4SF5 gene
STAT: Signal transducer and activator of transcription
T5ERM: TM4SF5-enriched microdomains
TM4SF5: transmembrane 4 L six family member 5
TM4SF5C-ter: the C-terminus of TM4SF5
