# Supplementary material for: TM4SF5 promotes metastatic behavior of cells in 3D extracellular matrix gels by reducing dependency on environmental cues
Source: Oncotarget. 2017 May 7;8(48):83480–94. doi: 10.18632/oncotarget.17644 (PMC5663530; doi:10.18632/oncotarget.17644)
Supplement: Supplementary file 1 [file oncotarget-08-83480-s001.pdf]

TM4SF5 promotes metastatic behavior of cells in 3D extracellular matrix gels by reducing dependency on environmental cues

Supplementary Materials

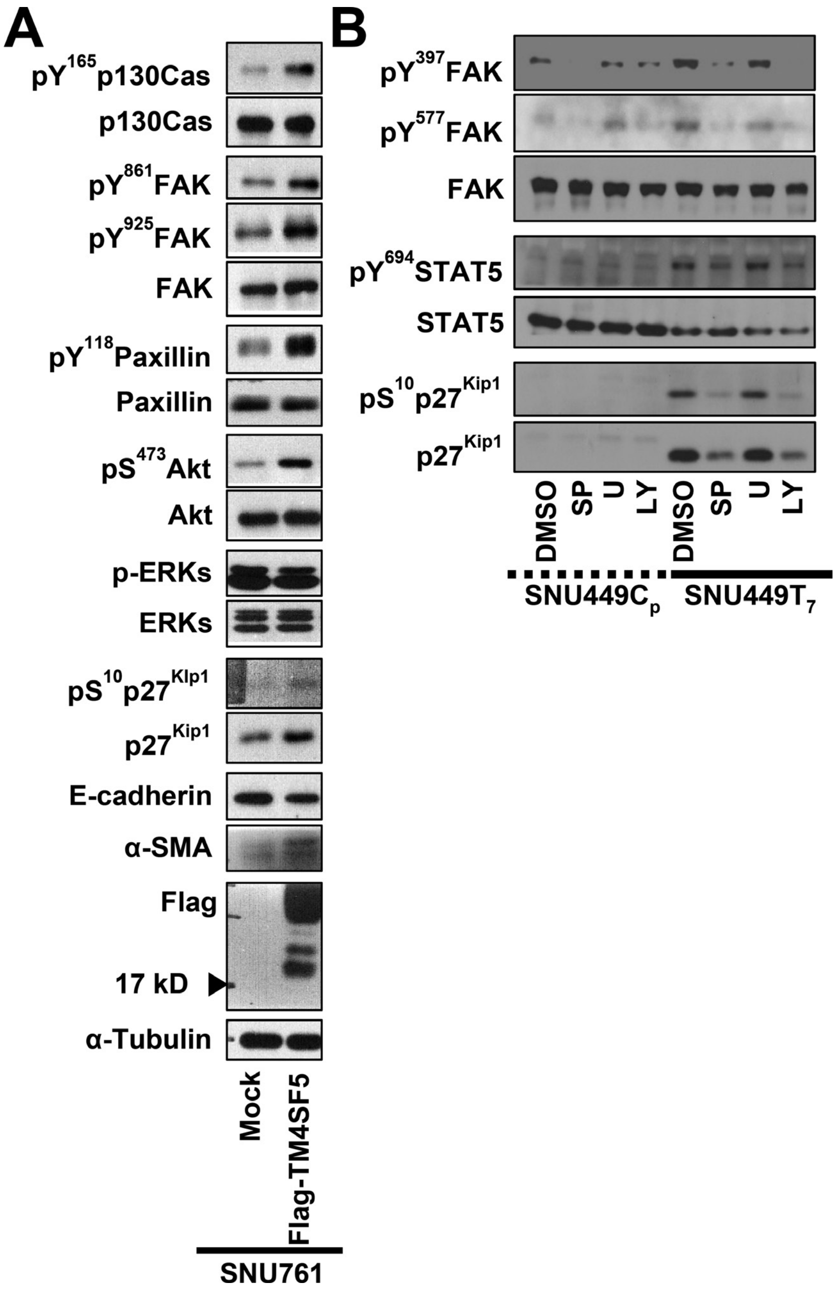

Supplementary Figure 1: TM4SF5-mediated signaling activities. (**A**) Subconfluent cells stably expressing Flag-Mock or Flag-TM4SF5 in 2D normal culture condition were harvested for whole cell extracts, before standard Western blots for the indicated molecules. (**B**) Stable cells were embedded into 3D Matrigel in the presence of DMSO, SP600125 (JNK inhibitor, 20 μM), U0125 (MEK/ERKs inhibitor, 20 μM), or LY294002 (PI3K/Akt inhibitor, 20 μM) for 24 h. Then the cells were harvested and processed for immunoblottings for the indicated molecules. Data shown represent 3 isolated experiments.

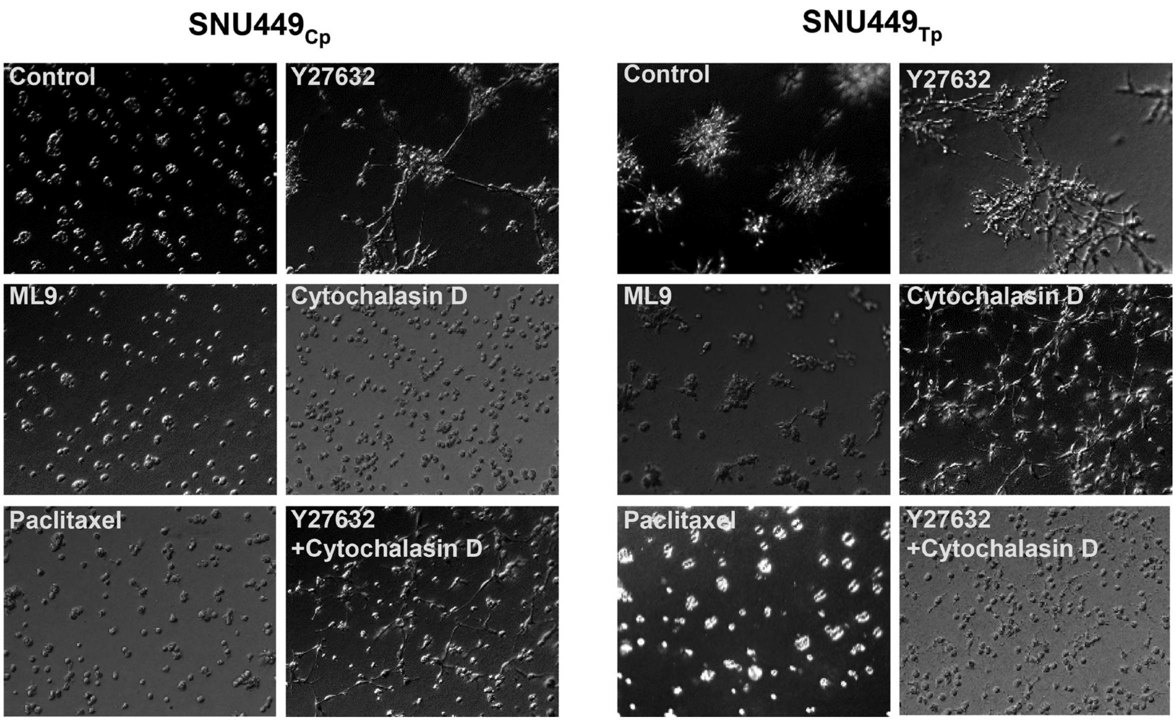

Supplementary Figure 2: Invasive foci formation of cells in 3D Matrigel depended on cytoskeletal integrity. Cells were embedded into 3D Matrigel. Diverse pharmacological inhibitors were included into the Matrigel during embedding, before live imaging for 17 h. Cells were also treated with DMSO (Control), Y27632 (a specific ROCK inhibitor), ML9 (a specific MLCK inhibitor), cytochalasin D (a F-actin destabilizing factor), Paclitaxel (a microtubule destabilizing factor), or Y27632 and Cytochalasin D during embedding, before live imaging. The representative end point images were shown.

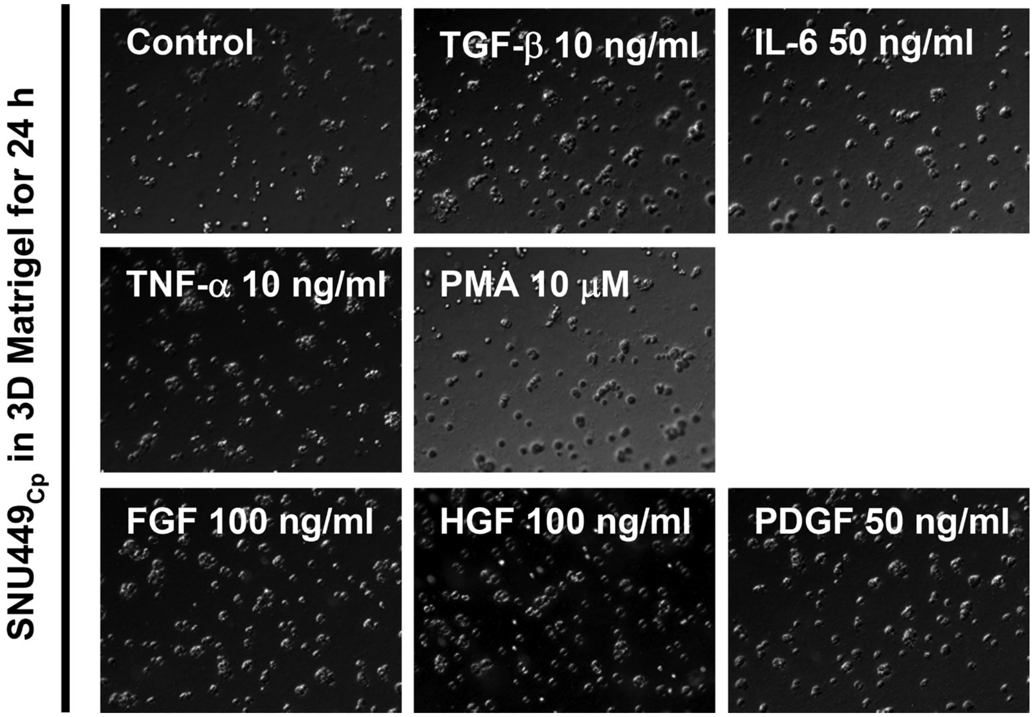

Supplementary Figure 3: SNU449$_{Cp}$ cells embedded into 3D matrigel slightly responded to TGFβ1, but not to other soluble factors, for the foci-forming tendency. SNU449$_{Cp}$ cells were embedded into 3D Matrigel without (Control) or with diverse soluble factors at the indicated concentrations for 24 h. Representative end point images were shown for each condition. Data shown represent 3 independent experiments.

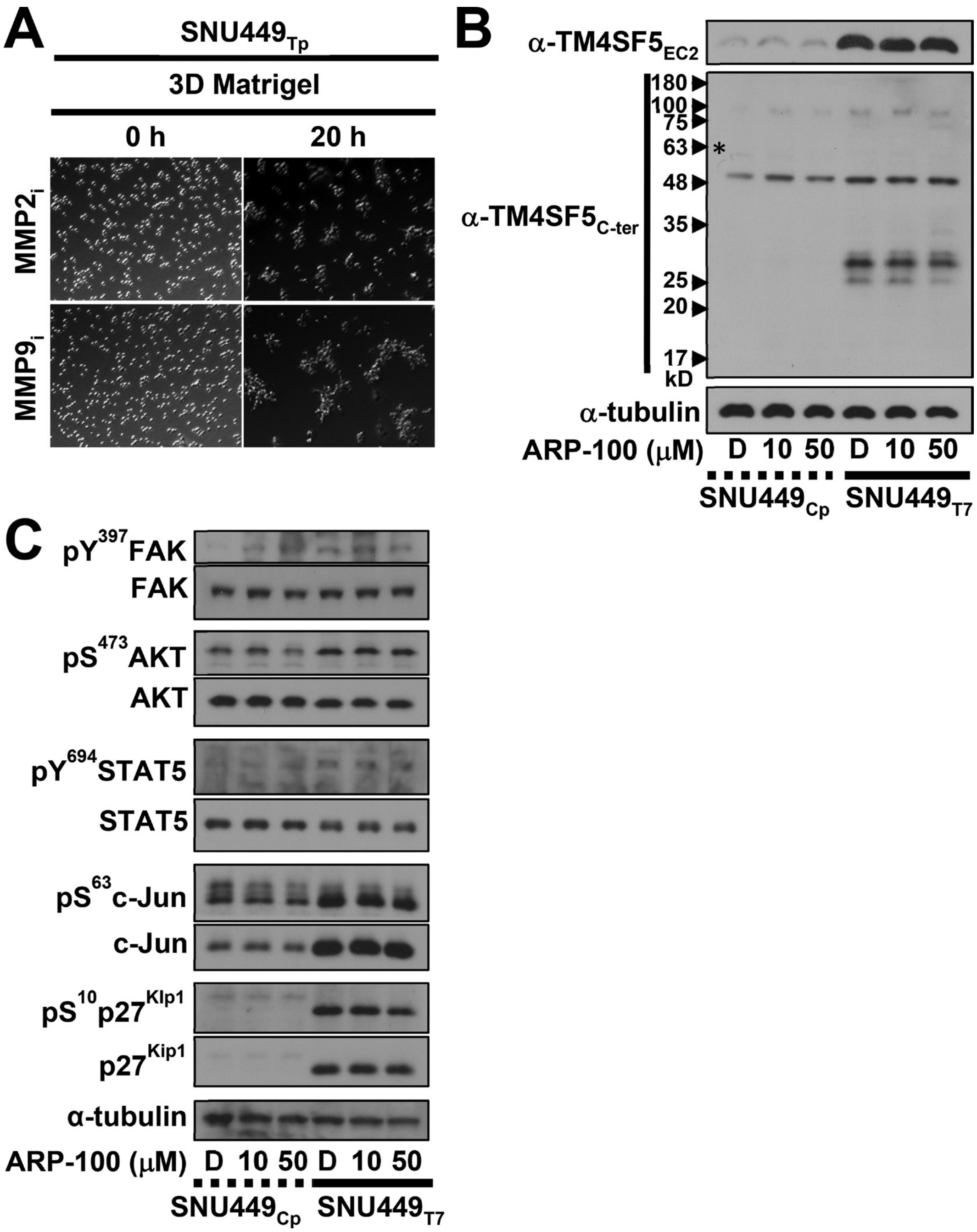

Supplementary Figure 4: Extracellular MMP2 activity was important for the TM4SF5-mediated invasive foci formation in 3D Matrigel. Cells were embedded into 3D Matrigel. During embedding, diverse pharmacological inhibitors, antibodies, or beads were included into the Matrigel. Then the cells were live imaged for 24 h, and cells were eventually harvested for whole cell lysates, before standard Western blots for the indicated molecules. $MMP2_i$ and $MMP9_i$ or ARP-100 (a specific MMP2 inhibitor) were treated. * in (B) depicts nonspecific bands.

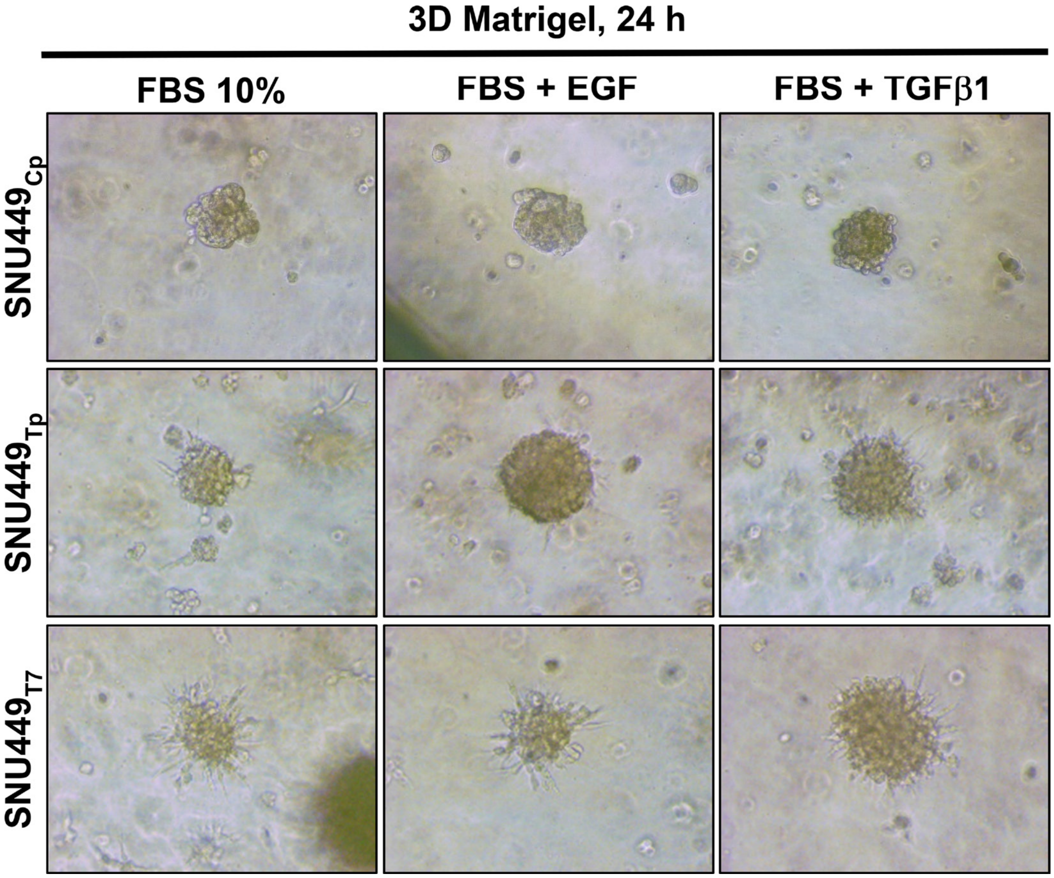

Supplementary Figure 5: TM4SF5 expression-dependent outgrowth from the spheroids embedded in 3D Matrigel in the presence of growth factors. SNU449 cells ($SNU449_{Cp}$ lacking TM4SF5 and $SNU449_{Tp}$ or $SNU449_{Tp}$ cell clones expressing TM4SF5) were embedded in 3D Matrigel in the presence of FBS 10%, FBS 10% plus EGF (50 ng/ml), or FBS plus TGFβ1 (10 ng/ml) for 24 with live imaging. The representative end point snap images were shown. Data shown represent 3 independent experiments.

Supplementary Movie 1: Related to Figure 1D. SNU449$_{Cp}$ cells embedded in 3D Matrigel. Live images (20 min/frame for 20 h) of the cells were saved using time-lapse microscopy (Olympus, IX81-ZDC). The microscope was equipped with a Lab-Tek 8 well chamber (NUNC), and an environmental chamber mounted on the microscope maintained constant conditions of 37°C, 5% CO_2, and 95% humidity. See Supplementary_Movie_1

Supplementary Movie 2: Related to Figure 1D. SNU449$_{Tp}$ cells embedded in 3D Matrigel. Live images (20 min/frame for 20 h) of the cells were saved using time-lapse microscopy (Olympus, IX81-ZDC). The microscope was equipped with a Lab-Tek 8 well chamber (NUNC), and an environmental chamber mounted on the microscope maintained constant conditions of 37°C, 5% CO_2, and 95% humidity. See Supplementary_Movie_2

Supplementary Movie 3: Related to Figure 2E. SNU449$_{Cp}$ cells embedded in 3D Matrigel in the presence of ROCK inhibitor Y27632 treatment. Live images (20 min/frame for 17 h) of the cells were saved using time-lapse microscopy (Olympus, IX81-ZDC). The microscope was equipped with a Lab-Tek 8 well chamber (NUNC), and an environmental chamber mounted on the microscope maintained constant conditions of 37°C, 5% CO_2, and 95% humidity. See Supplementary_Movie_3

Supplementary Movie 4: Related to Figure 4A. SNU449$_{Cp}$ and SNU449$_{Tp}$ cells at 50:50 ratio embedded in 3D Matrigel. Live fluorescent images (30 min/frame for 5 h) of the cells labelled with CellTracker Green CMFDA (for SNU449$_{Cp}$) and Red CMTPX (for SNU449$_{Tp}$) were saved using time-lapse microscopy (Olympus, IX81-ZDC). The microscope was equipped with a Lab-Tek 8 well chamber (NUNC), and an environmental chamber mounted on the microscope maintained constant conditions of 37°C, 5% CO_2, and 95% humidity. See Supplementary_Movie_4

Supplementary Movie 5: Related to Figure 4C. SNU449$_{Tp}$ cells embedded in 3D Matrigel and collagen I gels. Live images (30 min/frame for 24 h) of the cells were saved using time-lapse microscopy (Olympus, IX81-ZDC). The microscope was equipped with a Lab-Tek 8 well chamber (NUNC), and an environmental chamber mounted on the microscope maintained constant conditions of 37°C, 5% CO_2, and 95% humidity. See Supplementary_Movie_5

Supplementary Movie 6: Related to Figure 4C. SNU449$_{Cp}$ cells embedded in 3D Matrigel and collagen I gels in the presence of EGF treatment. Live images (30 min/frame for 24 h) of the cells were saved using time-lapse microscopy (Olympus, IX81-ZDC). The microscope was equipped with a Lab-Tek 8 well chamber (NUNC), and an environmental chamber mounted on the microscope maintained constant conditions of 37°C, 5% CO_2, and 95% humidity. See Supplementary_Movie_6

Supplementary Movie 7: Related to Figure 5E. SNU449$_{Cp}$ spheroids embedded in 3D collagen I gel. Live images (30 min/frame for 30 h) of the spheroids were saved using time-lapse microscopy (Olympus, IX81-ZDC). The microscope was equipped with a Lab-Tek 8 well chamber (NUNC), and an environmental chamber mounted on the microscope maintained constant conditions of 37°C, 5% CO_2, and 95% humidity. See Supplementary_Movie_7

Supplementary Movie 8: Related to Figure 5E. SNU449$_{Tp}$ spheroids embedded in 3D collagen I gel. Live images (30 min/frame for 30 h) of the spheroids were saved using time-lapse microscopy (Olympus, IX81-ZDC). The microscope was equipped with a Lab-Tek 8 well chamber (NUNC), and an environmental chamber mounted on the microscope maintained constant conditions of 37°C, 5% CO_2, and 95% humidity. See Supplementary_Movie_8
